# The Metabolism of Creatinine and Its Usefulness to Evaluate Kidney Function and Body Composition in Clinical Practice

**DOI:** 10.3390/biom15010041

**Published:** 2025-01-01

**Authors:** Marcela Ávila, Mariana G. Mora Sánchez, Alma Sofía Bernal Amador, Ramón Paniagua

**Affiliations:** Unidad de Investigación Médica en Enfermedades Nefrológicas, Hospital de Especialidades, CMN SXXI, Instituto Mexicano del Seguro Social, Ciudad de México 06720, Mexico; mariana.gmora99@gmail.com (M.G.M.S.); sofiabernalama@gmail.com (A.S.B.A.)

**Keywords:** creatinine metabolism, glomerular filtration rate, muscle mass markers, cystatin C, chronic kidney disease, creatinine kinetics, CKD biomarkers

## Abstract

Serum creatinine levels are the most used clinical marker to estimate renal function as the glomerular function rate because it is simple, fast, and inexpensive. However, creatinine has limitations, as its levels can be influenced by factors such as advanced age, physical activity, protein-rich diets, male gender, medications, and ethnicity. Serum cystatin C and its combination with serum creatinine may serve as an alternative since these factors do not affect it. Most creatinine synthesis occurs in the muscles, making it a valuable marker for assessing lean body mass within body composition. This measurement is crucial for evaluating and monitoring nutritional status in patients with chronic kidney disease. This review aimed to discuss the literature on creatinine metabolism, its advantages and disadvantages in assessing renal function, and its utility in measuring lean body mass. The variability in the creatinine generation rate among individuals should be considered when assessing the glomerular function rate.

## 1. Introduction

Creatinine (Cr) was discovered by French chemist Michel Eugène Chevreul in 1832 while investigating the composition of the skeletal muscles. He named the component “creatinine”, derived from the Greek word *kreas*, meaning meat.

Cr, chemically known as α-methyl guanidinoacetic acid, is a substance that appears as white crystalline particles in its pure state. It has a molecular weight of 113.1 Daltons and behaves as a cation in aqueous solutions.

It is consistently produced as part of normal muscle cells, serving as the final product of creatine (Crn) and phosphocreatine (PCrn) metabolism, and dietary protein intake, and eliminated by extrarenal degradation and urinary excretion [1].

Serum creatinine (SCr) concentration is the most used clinical indicator for assessing the glomerular function rate (GFR). Its widespread use is based on the correlation of its concentration with the precise measurement of the GFR using the clearance of substances like inulin (the gold standard) [2]. The broad availability, technical simplicity, and low cost of Cr measurement contribute to its use in routine renal function assessment.

Despite these practical advantages, Cr has limitations as a GFR marker. These include the imprecision of conventional quantification methods, which rely on chemical reactions (Jaffé) prone to interference from other molecules. Certain medications, such as cimetidine, trimethoprim, and abemaciclib, inhibit the tubular secretion of Cr by competing for secretion pathways, thereby increasing SCr levels and leading to erroneous estimates of the GFR (decreased) [3]. Additionally, Cr levels vary with muscle mass, dietary protein intake, and creatine supplementation. Levels tend to decrease with aging, in females, and in individuals of White ethnicity [4].

In patients with chronic kidney disease (CKD), it is important to determine lean body mass (LBM) as part of nutritional status monitoring. Methods such as Bioimpedance Analysis (BIA) and Dual-Energy X-ray Absorptiometry (DEXA) are used, but these are sophisticated techniques with limited accessibility in many hospitals due to their cost and the need for specialized personnel and equipment. Consequently, the Cr index (CI) or Cr kinetic where SCr is used has been a marker of LBM [5].

The objective of this review was to describe Cr metabolism, the factors influencing its serum concentration, and the considerations required for interpreting Cr concentration as a marker of the GFR and muscle mass in healthy individuals and CKD patients.

## 2. Creatinine Synthesis

Cr is distributed through total body water (TBW). SCr is formed through a spontaneous non-enzymatic anhydration of Crn in muscle cells. This conversion of Crn to Cr is influenced by pH and temperature; this process is carried out at a constant rate (1% of body Crn and 2.6% of PCrn per day is changed to Cr) [6].

**Figure 1:**
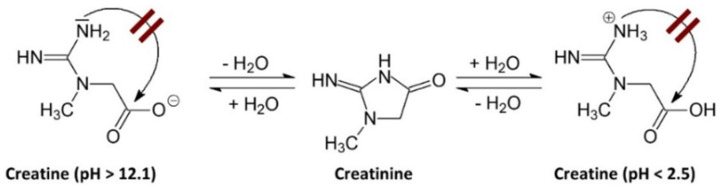
Effect of pH on creatinine stability. A pH above 12.1 induces deprotonation of the acidic group, hindering intramolecular cyclization towards Cr. Conversely, when the pH is below 2.5, the amide group of the Crn molecule protonates, which also prevents intramolecular cyclization towards Cr [7].

The degradation of Crn can be limited or may not occur in environments with very low or very high pH. A pH above 12.1 favors the deprotonation of the acid group, which hinders the intramolecular cyclization to form Cr. Conversely, when the pH is below 2.5, the amide functional group of the Crn molecule is protonated, also preventing intramolecular cyclization [7], as shown in Figure 1.

## 3. Creatine Sources

**Figure 2:**
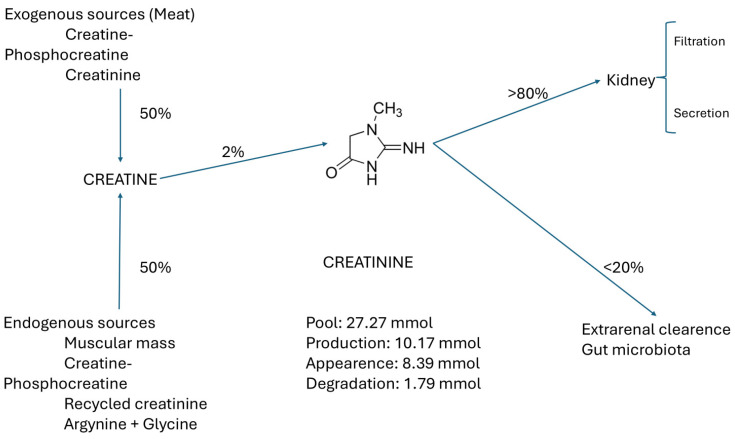
Main sources and pathways of creatinine elimination.

Crn comes from two main sources: 50% exogenous, from food, phosphate creatine (PCr), and commercial Crn, and 50% endogenous (from muscle, Crn, creatine phosphate (PCr), recycled Cr, and glycine + arginine), of which only 2% is converted to Cr. More than 80% of SCr is filtered and secreted in the kidney. The remaining 20% is cleared through the gut microbiota, as shown in Figure 2 [8].

### 3.1. Creatine from Exogenous Sources

#### 3.1.1. In Foods

Exogenous Crn primarily comes from the diet and its processing. Some of the main foods where Crn can be found are shown in Table 1 [9].

#### 3.1.2. Cooked Meat

The cooking process is known to convert a fraction of the Crn contained in meat into Cr [9]. Around 20–30 years ago, some studies demonstrated that the intake of cooked meat (225–500 g) significantly increased the concentration of SCr [10,11].

Preiss et al. conducted a study that demonstrated that the effect of the intake of cooked meat compared to uncooked meat increases SCr by 20.5 mmol/L during the first 1 to 2 h and by 18.5 mmol/L between 3 and 4 h postprandial in healthy subjects and in stage 3 chronic CKD. Furthermore, the estimated glomerular function rate (eGFR) calculated with SCr decreased by 24.5 mL/min/1.73 m^2^ between the first 1 and 2 h and by 20 mL/min/1.73 m^2^ at 3–4 h postprandial, calculated using three methods [12]. Misclassification of CKD is possible if measurements are made after meals containing cooked meat.

Since meat is the primary source of exogenous Crn, vegans and vegetarians have a reduced intake of this essential compound, resulting in decreased muscle Crn stores, so supplementation helps to compensate for this reduction [13].

#### 3.1.3. Creatine as a Dietary Supplement

Crn is an ergogenic supplement that has been used by athletes to increase strength gains. Various forms of commercial Crn exist; however, Crn monohydrate has been the most extensively studied, and its formulation has shown benefits in short-duration, high-intensity weightlifting, as well as in cycling [14]. In a review on the utility of Crn, it was found that its supplementation may improve post-exercise recovery, including injury prevention, thermoregulation, rehabilitation, and neuroprotection following a concussion in animal models, as well as enhancing functional capacity in individuals with spinal cord injury [15]. Additionally, its clinical applications have been investigated in neurodegenerative diseases such as muscular dystrophy, Parkinson’s disease, and Huntington’s disease, as well as in diabetes, osteoarthritis, fibromyalgia, aging, cerebral and cardiac ischemia, and depression in adolescents and pregnant women [16].

Research conducted in various age groups that received Crn supplements at doses of 0.3 to 0.8 g/kg/day for up to 5 years has demonstrated that supplementation with Crn does not pose negative health risks. Moreover, these studies suggest that Crn may provide several benefits for both general health and physical performance [16].

Burke et al. found that supplementation with Crn helped to improve low Crn reserves in the muscles of vegetarians, who showed more significant increases in fat-free mass (FFM), maximal strength, and type II muscle fiber area compared to those following an omnivorous diet [17].

### 3.2. Creatine from Endogenous Sources

**Figure 3:**
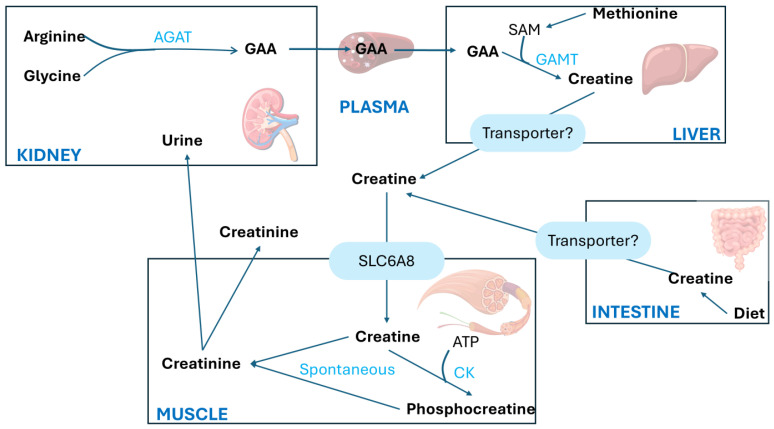
Creatine metabolism adapted from Ref. [18]. AGAT, arginine/glycine amidinotransferase; CK, creatine kinase; GAA, guanidinoacetate; GAMT, guanidinoacetate methyltransferase; SAM, S-adenosylmethionine; SLC6A8, creatine transporter [18].

The synthesis of Crn begins in the kidneys primarily with the transfer of the amino group from arginine (Arg) to glycine (Gly), producing L-ornithine and guanidinoacetic acid (GAA). This first step is catalyzed by the enzyme L-arginine aminotransferase (AGAT). The second step involves the methylation of guanidinoacetic acid through the action of S-adenosyl-L-methionine/N-guanidoacetate methyltransferase (GAMT), which transfers a methyl group from S-adenosylmethionine (SAM), thereby forming Crn, as shown in Figure 3.

Crn is synthesized mainly in the liver and is transported into the bloodstream via a specific transporter to the muscles, which contains approximately 98% of the total body Crn reserves. The highest concentrations of Cr and PCr are found in the skeletal muscle, heart, sperm, and photoreceptor cells of the retina in mammals.

A large amount of PCr is available in fast-twitch skeletal muscles for the immediate regeneration of hydrolyzed adenosine triphosphate (ATP) during short periods of intense work [8]. The synthesized Crn reaches the designated tissues through the transport of blood vessels and intracellular transport mediated by a neurotransmitter called the Crn transporter, which depends on chloride and sodium ions.

**Figure 4:**
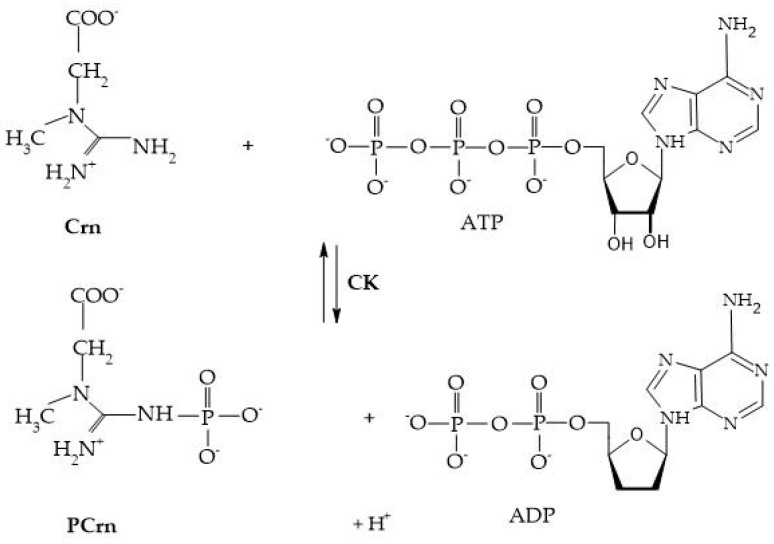
Creatine kinase reaction.

Crn and its phosphorylated form are crucial for maintaining ATP reserves in cells with high energy demands, such as myocytes, cardiomyocytes, hepatocytes, enterocytes, inner ear cells, renal cells, sperm cells, and photoreceptors. The enzyme creatine kinase (CK) initiates the reversible transfer reaction after reaching the intracellular space, leading to the creation of PCr [8], as shown in Figure 4.

## 4. Creatinine Metabolic Pool

Basic indicators of Cr metabolism in a patient with CKD on hemodialysis (HD). Pool: 27.27 mmol, production: 10.17 mmol, appearance: 8.39 mmol, and degradation: 1.79 mmol.

The content of Cr in the tissues and its concentration in the extracellular fluid (ECW) (and serum) remain stable unless there is acute muscular destruction, urinary tract obstruction, or acute kidney failure. The pool of Cr has two access pathways: (a) the endogenous generation in the muscles as the final product of the Crn metabolism through recycling, and (b) the intake of Cr or its precursor Crn in meat or supplements. Cr is eliminated by two pathways, primarily by the kidneys through filtration and secretion, and to a lesser extent by metabolism in the gut microbiota [4], as seen in Figure 2.

CK, creatine kinase; PCrn, phosphocreatine; Crn, creatine; ATP, adenosine triphosphate; and ADP, adenosine diphosphate [8].

### 4.1. Renal Clearence

Cr, being a small molecule of low molecular weight and not bound to proteins, distributes throughout the total body water and is filtered by the glomerulus. However, the estimation of its serum concentration can be significantly influenced by factors beyond glomerular filtration, such as extrarenal elimination and tubular secretion. Glomerular filtration is the initial step for its elimination from the body. Thus, SCr is predominantly excreted by glomerular ultrafiltration, so when GFR decreases, Cr accumulates.

The glomerular fenestrated endothelium allows small molecules like Cr to pass freely through its pores. The pores of this endothelium are large enough to permit the passage of water and small solutes but small enough to retain blood cells and larger proteins. The glomerular basement membrane filters molecules based on size and electrical charge, and although Cr is relatively small, its passage through the basement membrane is also facilitated by its solubility in water. Podocyte cells surround the glomerular capillaries, and their extensions (called pedicels) form filtration slits that allow Cr to pass freely into the Bowman’s capsule space [19]. Once Cr crosses the glomerular barrier, it becomes part of the glomerular filtrate and flows into the proximal tubules of the nephron.

The first studies on the secretion of Cr indicated that it accounted for 10% to 40% of urinary Cr excretion (UCrE) in healthy subjects [20]. More recently, studies such as the one by Imamura et al. indicated that tubular secretion contributes significantly to renal Cr elimination, accounting for 30% to 60% of the total elimination of Cr [21].

Zhang et al. analyzed data from different cohorts of the Modification of Diet in Renal Disease (MDRD) study, the African American Study of Kidney Disease and Hypertension (AASK), and the Mayo Clinic to determine the correlation between creatinine clearance (CrCl) and estimated glomerular filtration rate (eGFR) in patients with varying degrees of renal function. The results indicated that the CrCl/eGFR ratio is increased in patients with reduced eGFR due to the increased tubular secretion of Cr in this group, which is not accounted for; thus, in these cases, CrCl tends to overestimate eGFR [22].

### 4.2. Creatinine Transporters in Renal Proximal Tubules

Urakami et al. demonstrated that in the renal proximal tubules, the Basolateral Organic Cation Transporters hOAT1 (SLC22A6), hOAT3 (SLC22A8), and hOCT2 (SLC22A2) are primarily expressed in the basolateral membrane and play a crucial role in the uptake of organic compounds from the blood, with Cr being an endogenous substrate of hOCT2 [23]. Additionally, Tanihara et al. revealed that the proteins MATE1 and MATE2-K, which are responsible for the exchange of protons and organic cations in the brush border membrane of the proximal tubules, also accept Cr as a substrate, facilitating efficient transcellular transport in conjunction with hOCT2 [24].

And since various drugs act as substrates for these transporters, they interfere with the tubular secretion of Cr, affecting SCr concentrations, as will be discussed later.

It is important to note that Jones and Burnett previously estimated that 16-66% of synthesized Cr has extrarenal degradation routs by intestinal microbiota in patients with decreased renal function [25].

### 4.3. Extrarenal Degradation of Creatinine by Gut Microbiota

Bacteria and fungi capable of degrading Crn and Cr through metabolic pathways have been identified in the droppings of chickens and pigeons, as well as in human urine, feces, and the bacterial flora of the human colon. These bacteria may be particularly relevant in kidney disease. In uremic patients with significantly elevated SCr levels, it is believed that Cr diffuses into the intestinal tract, where it induces the activity of creatininase, creatinase, and creatinine deaminase, ultimately resulting in the breakdown of part of the body’s Cr reserves and its partial recycling.

**Figure 5:**
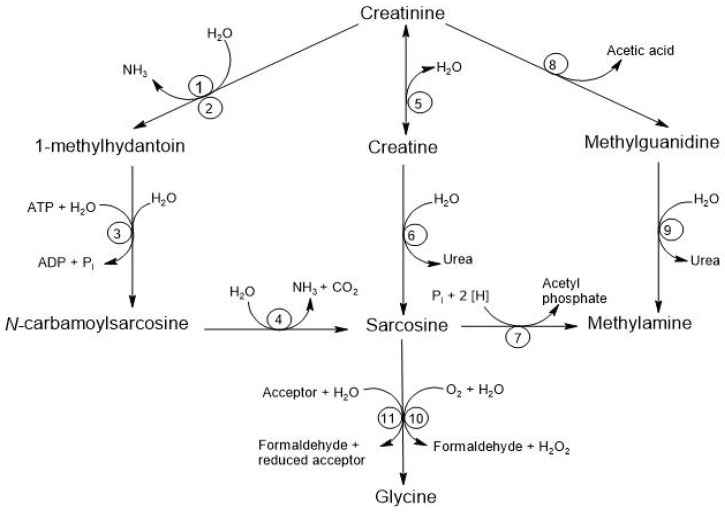
Degradation of creatinine by the intestinal microbiota. The respective enzymes are denoted by numbers: (1) creatinine amidohydrolase (creatinine deaminase; EC 3.5.4.21); (2) cytosine amidohydrolase (cytosine deaminase; EC 3.5.4.1); (3) 1-methylhydantoin amidohydrolase [ATP-dependent (EC 3.5.2.14) or ATP-independent]; (4) N-carbamoyl-sarcosine amidohydrolase (EC 3.5.1.59); (5) creatinine amidohydrolase (creatinase; EC 3.5.2.10); (6) creatine amidinohydrolase (creatinase; EC 3.5.3.3); (7) sarcosine reductase (EC 1.4.4.-); (8) not yet characterized; (9) methylguanidine amidinohydrolase (EC 3.5.3.16); (10) sarcosine oxidase (EC 1.5.3.1); (11) sarcosine dehydrogenase (EC 1.5.99.1) or dimethylglycine dehydrogenase (EC 1.5.99.2) [8].

There are at least four alternative pathways for the microbial degradation of Cr. The first pathway is the degradation to 1-methylhydantoin, in which certain microorganisms, such as *Bacillus*, *Clostridium*, and *Escherichia*, degrade Cr into 1-methylhydantoin and ammonia, using Cr as a nitrogen source. The second pathway is the degradation to N-carbamoyl-sarcosine, where strains like *Pseudomonas* and *Brevibacterium* convert 1-methylhydantoin into N-carbamoyl-sarcosine and sarcosine. The third pathway involves degradation to Crn where different species, including *Alcaligenes*, *Arthrobacter*, and *Tissierella*, use creatininase to convert Cr into Crn, which is subsequently metabolized to urea and sarcosine through the action of creatinase. Finally, the fourth pathway is the degradation to methylguanidine, where *Pseudomonas stutzeri* has been observed to convert Cr into methylguanidine and acetic acid, and this methylguanidine can be broken down by certain *Alcaligenes* strains, producing methylamine and urea [8]. See Figure 5.

Understanding creatinine metabolism is important because it allows us to understand the pathophysiological mechanisms involved in its impact as a marker of renal function and prevent the misinterpretation of eGFR. Cr reflects not only renal function but also the generation, intake, and metabolism of Cr. One important aspect is its interaction with some medicaments. Cr may modify renal clearance and increase the medications’ half-life. Medication may interfere with Cr secretion and lead to a sub-estimation of renal function.

## 5. Factors Affecting Serum Creatinine Concentration

**Figure 6:**
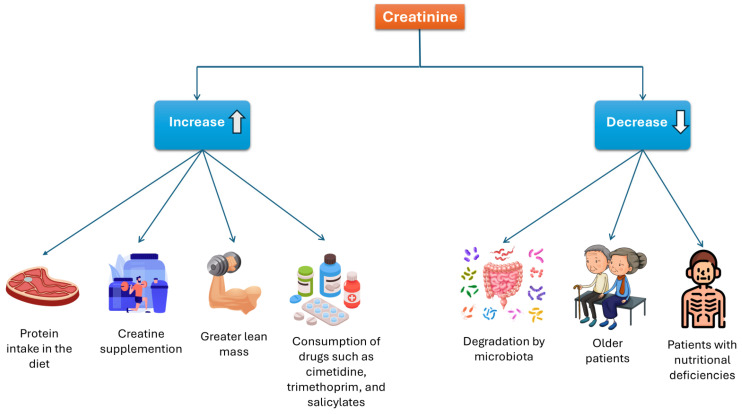
Factors that influence serum creatinine concentration.

As mentioned previously, Cr levels can be affected by various factors. Some may increase Cr levels, including a high-protein diet, Crn supplementation, muscle mass, and certain medications. Conversely, factors that can decrease Cr concentrations include degradation by intestinal microbiota, advanced age, and nutritional deficiencies. See Figure 6.

### 5.1. Drugs That Increase Serum Creatinine Concentrations

#### 5.1.1. Cimetidine

Cimetidine is an H2 receptor antagonist that inhibits gastric acid secretion at a dose of 1.6 g/day. This medication ensures SCr concentrations at an average of 15% in patients with normal renal function [26,27]. High single doses of cimetidine, such as 300 mg intravenously or 800 mg orally, resulted in a reduction in both endogenous and exogenous CrCl (14C-creatinine) by 20 ± 30% in healthy volunteers. This effect is attributed to a decrease in UCrE and an accumulation of SCr until a new steady state is reached. However, there were no concomitant changes in GFR when measured with inulin [3,28].

Cimetidine has a high affinity for the organic cation transporter OCT2 and the multidrug and toxin extrusion transporters (MATE) in the luminal membrane of the proximal tubules, similar to Cr [29]. Therefore, cimetidine inhibits the tubular secretion of Cr, meaning that the absorption of cimetidine blocks the tubular secretion of Cr, resulting in a CrCl close to or identical to the clearance measured with Iohexol, which is also considered a gold standard for measuring glomerular filtration [30].

Therefore, the blockage of the tubular secretion of Cr with cimetidine has proven to be a useful tool for measuring the glomerular filtration rate in kidney transplant recipients with SCr concentrations less than 2.5 mg/L. It has been observed that the clearance calculated using the MDRD formula was similar to that obtained after the administration of cimetidine, reinforcing its viability as an alternative method in this context [31].

#### 5.1.2. Trimethoprim

Trimethoprim is an antibacterial that inhibits the enzyme dihydrofolate reductase [32]. Clinical studies have demonstrated that at therapeutic doses, it elevates SCr concentrations by 15% to 30% and reduces CrCl by 20% to 25%, without affecting the glomerular filtration measured with iodothalamate [33,34].

This effect of trimethoprim is explained by the inhibition of Na/K ATPase present in the basal membrane of the epithelial cells of the distal tubule, as well as the OCT2 transporter and the MATE 1/2-K transporters in the proximal tubule [35,36].

#### 5.1.3. Salicylates

Salicylates can induce an increase in SCr concentration by altering the binding of Cr to serum proteins or competitively inhibiting the tubular secretion of Cr under certain conditions (salt depletion, advanced age, liver cirrhosis, renal diseases, and renal insufficiency) [37,38,39].

#### 5.1.4. Abemaciclib

Abemaciclib is a cyclin-dependent kinase 4 and 6 inhibitor indicated for the treatment of metastatic breast cancer. This medication has been shown to increase SCr concentrations by 10–40%, with peak Cr levels occurring 10–12 h after administration. However, this increase is reversible and demonstrates a gradual decrease over time [40,41].

The GFR calculated using the CKD-EPI (Chronic Kidney Disease Epidemiology Collaboration) formula shows a decrease following the administration of abemaciclib, but there are no changes in GFR measured with Iohexol or in the eGFR calculated from serum concentrations of CysC. Furthermore, no significant changes were observed in urinary concentrations of neutrophil gelatinase-associated lipocalin (NGAL) and kidney injury molecule 1 (KIM-1), which are biomarkers of renal damage, normalized to Cr.

These data, along with the changes observed in the pharmacokinetics of metformin, suggest that the elevations in SCr concentrations observed in clinical studies of abemaciclib are due to a reversible inhibition of the renal tubular secretion of Cr rather than acute kidney injury [42].

#### 5.1.5. Integrase Inhibitors

Drugs used for the treatment of HIV, such as Dolutegravir (DTG), Raltegravir (RAL), and Elvitegravir (EVG), have been associated with an increase in SCr concentrations. In the VIKING study, which compared the efficacy of DTG versus RAL, the administration of DTG resulted in an increase in Cr levels between 0.084 and 0.105 mg/dL, with this effect being more evident on the 6th and 8th days after administration, reaching a plateau by week 4 without further progression [43].

In the SPRING-2 study, patients treated with DTG showed an increase of 14.6 μmol/L in SCr concentrations after 96 weeks of treatment. Similarly, an increase in Cr of 8.2 μmol/L was observed following the administration of RAL. Additionally, the change in CrCl calculated using the Cockcroft–Gault formula was −19.6 mL/min in the DTG group and −9.3 mL/min in the RAL group, both at week 96 [44]. Furthermore, in additional studies of patients treated with DTG, either alone or in combination with Tenofovir disoproxil fumarate (TDF), renal function estimated by Cr (eGFRCr) and by cystatin C (eGFRCys) was compared. The results indicated that treatment with DTG was associated with a significant increase in Cr and a reduction in eGFRCr, while the cystatin C concentrations and eGFR_Cys remained unchanged [45,46].

This pattern, where there is a rapid but small increase in SCr concentrations that subsequently levels off without further progression, is typical of the blockade of Cr secretion via OCT2 [47].

**Figure 7:**
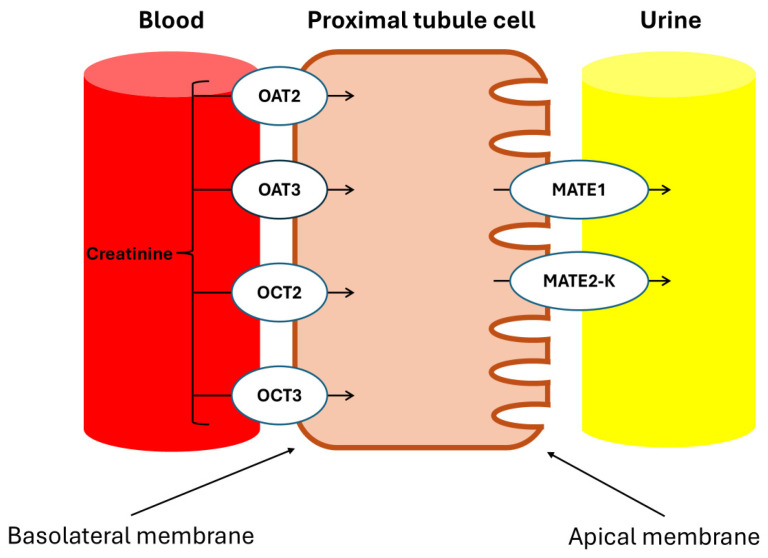
Drug transporters in proximal kidney cells. OAT2: organic anion transporter 2; OAT3: organic anion transporter 3; OCT2: organic cation transporter 2; OCT3: organic cation transporter 3; MATE1: multidrug and toxin extrusion protein 1; MATE2-K: multidrug and toxin extrusion protein 2, kidney-specific. The tubular secretion of creatinine involves basolateral uptake through the OCT2 and OCT3 transporters for organic cations, and the OAT2 and OAT3 transporters for organic anions. Subsequently, the apical elimination of Cr into the tubular lumen is facilitated by the MATE1 and MATE2-K transporters, which regulate its excretion from the proximal tubular cells.

These transporters involved in the secretion of Cr are illustrated in Figure 7.

#### 5.1.6. Glucocorticoids

Glucocorticoids are catabolic hormones from the corticosteroid family, commonly used to suppress, prevent, or reduce immune responses [48]. Due to the catabolic effect of glucocorticoids, there is a release of Crn at the muscular level, which is subsequently converted into Cr, leading to increased serum levels as well as urinary excretion [49].

### 5.2. Sex

A longitudinal study evaluated urinary Cr excretion and CrCl in men and women. The results showed that men excreted 33% more Cr daily than women, even after adjusting for weight. This difference was attributed to men’s greater lean body mass, contributing to higher production and, consequently, the elimination of Cr. Regarding SCr concentrations, men exhibited levels that were 21% higher compared to women [50].

The results of the Third National Health and Nutrition Examination Survey (NHANES) conducted in the United States indicated that women had Cr levels approximately 22% lower than men [51].

### 5.3. Race and Ethnicity

In the study mentioned above, James et al. reported that Black subjects had a Cr production rate 5% higher compared to White individuals. This difference was attributed to greater muscle mass, rather than a higher protein intake in their diet, as potassium excretion was lower in this group [50].

Hsu C-Y et al. found a 10.7% increase in SCr concentrations in comparison to non-Black subjects [52]. Similarly, this was attributed to a higher muscle mass in this population group [53]. In this same study, muscle mass and SCr were evaluated in HD patients. It was found that SCr values for Black, Asian, and Hispanic patients were higher than those for non-Hispanic White patients, with differences of more than 1.68 mg/dL, 1.61 mg/dL, and 0.83 mg/dL, respectively. However, these differences among the various groups persisted even after adjusting for intracellular water.

Black patients had SCr concentrations very similar to those of Asian patients, with a minimal difference of only 0.03 mg/dL. However, the concentrations were significantly higher compared to Hispanic and non-Hispanic White patients. Unlike other studies, this study suggests that the higher SCr concentration may not be exclusively due to a greater amount of muscle mass [54].

Since these results challenge established knowledge, further studies are essential to clarify these findings and better understand the factors involved in SCr about muscle mass.

### 5.4. Physical Activity

Individuals with moderate/intense physical activity presented significantly higher SCr and albumin levels compared to those with sedentary or light physical activity, and higher urinary Cr excretion (UCrE) than sedentary individuals. There were no differences in serum CysC, urea, microalbuminuria, or measured CrCl between these groups. People with moderate/intense physical activity tended to lower CrCl and GFR (Cockcroft–Gault and MDRD equations) compared to sedentary individuals, though these differences did not reach statistical significance [55].

Individuals with higher levels of physical activity had lower body weight, BMI, waist circumference, and body fat content, as well as greater muscle mass compared to those who were sedentary or had light physical activity.

These results demonstrate that intense physical activity directly influences body composition, reducing body fat and increasing LBM. In contrast, serum CysC did not differ between groups. These findings further corroborate that CysC is not influenced by muscle mass [55].

Another study conducted by Beunders et al. showed a significant increase in SCr immediately after an exercise session, with concentrations rising from 58 ± 13 to 71 ± 11 µmol/L. The increase in SCr levels induced by exercise could imply a significant reduction in eGFR of −34 ± 33 mL/min/1.73 m^2^ [56].

### 5.5. Age

Kidney function and muscle mass decline progressively with age, the last commonly due to skeletal muscle atrophy. Serum Cr in patients with muscle mass loss might mask the loss of kidney function.

In patients over 65, a cutoff value of SCr of 1.7 mg/dL was used to identify renal insufficiency. It was found that 87.4% of patients with renal insufficiency confirmed by the Cockcroft–Gault formula to estimate GFR had SCr levels of 1.7 mg/dL or lower. These findings highlight the limitations of using SCr as a standalone marker for detecting renal insufficiency in older individuals [57].

Taking into account the aforementioned aspects, formulas that consider other parameters are recommended for a more accurate evaluation. Formulas to estimate GFR have recently eliminated the race factor to prevent race or ethnicity discrimination. Clinicians should be aware of which formula is used in their estimation.

## 6. Biochemical Measurement in the Laboratory

The normal range of SCr for adult men typically ranges from 0.74 to 1.35 mg/dL (65.4 to 119.3 μM), while for adult women, it ranges from 0.59 to 1.04 mg/dL (52.2 to 91.9 μM) [58]. The daily UCrE in healthy individuals ranges from 0.8 to 2.0 g/day. Elevated SCr levels, exceeding 1000 μM, can indicate renal dysfunction or muscle disorders.

Several conventional methods are available for measuring Cr levels, including colorimetric, enzymatic, and chromatographic techniques. While these methods are highly sensitive and selective, they also have drawbacks such as being time-consuming, requiring sample pre-treatment, high-cost instrumentation, and requiring skilled personnel to operate the equipment [59].

Colorimetric methods are based on the Jaffé reaction, which dates back to 1886. SCr reacts with picric acid in an alkaline medium, forming a red-colored complex at a wavelength between 510 and 520 nm. This method is simple, fast, and inexpensive.

One issue with this measurement is that, in addition to SCr, other positively charged molecules, such as proteins, glucose, acetoacetate, ascorbic acid, and uric acid, also react with picric acid as positive interferents. At the same time, there are also negative interferents, the most significant of which is bilirubin. Therefore, this technique has low specificity. In samples with elevated bilirubin levels, SCr values appear reduced because bilirubin in alkaline media oxidizes to biliverdin, forming a colorless compound that diminishes the color of the reaction. Non-creatinine chromogens can interfere with the assay, causing errors of up to 20% in normal individuals [1]. The Scr results may vary between laboratories; to validate these results and homogenize them, external quality control is necessary.

In children, particularly in the neonatal period, the Jaffé assay is more likely to be affected by non-creatinine chromogens in the sample, leading to a less accurate measurement. Additionally, the higher prevalence of jaundice and hemolyzed samples in this age group makes the results obtained less reliable [60].

### Enzymatic Measurement

The Cr present in the sample is converted to creatine Crn by the action of the enzyme Cr amidohydrolase. The resulting Crn is then hydrolyzed to sarcosine and urea through the action of the enzyme Crn amidinohydrolase. Next, sarcosine oxidase promotes the oxidative demethylation of sarcosine, producing glycine, formaldehyde, and hydrogen peroxide.

In the presence of peroxidase, the hydrogen peroxide formed reacts with N-ethyl-N-sulfopropyl-m-toluidine (ESPMT) and 4-aminoantipyrine, producing a quinoneimine with a maximum absorbance at 546 nm. The intensity of the color of the reaction product is directly proportional to the Cr concentration in the sample [61].

Another enzymatic method available for measuring Cr utilizes the enzyme Cr amidohydrolase. This method involves a four-step reaction process, ultimately measuring the decrease in NADH (Nicotinamide Adenine Dinucleotide) with a reading at 340 nm. Unlike the other enzymatic method, which relies on photometric readings outside the UV range, this method uses UV readings. The error in determining Cr impacts medical decisions based on guidelines. Standardizing Cr by increasing accuracy and reducing variation between laboratories helps prevent the incorrect classification and treatment of patients [61].

The estimation of GFR from SCr is adequate for diagnosing, staging, and monitoring CKD progression in most clinical circumstances. However, like all diagnostic tests, the interpretation is influenced by the test’s variable characteristics in selected clinical circumstances and the pre-test probability of disease. In particular, an isolated reduced GFR is more likely to be a false positive in otherwise healthy individuals than in those with risk factors for kidney disease or markers of renal damage [60].

Clinicians must be aware of the characteristics of the techniques used in Cr assessment and the potential consequences of estimating GFR. The presence of known factors that interfere with the Jaffe technique warrants the use of other alternative techniques or even other biomarkers.

## 7. Evaluation of Glomerular Filtration Rate

The GFR is measured through clearances, CrCl = (urine vol 24 h mL/1440 min) * (UCr (mg/dL)/SCr (mg/dL), and with endogenous or exogenous metabolites of glomerular filtration.

The recommendation for GFR evaluation, according to the 2024 Kidney Disease Improving Global Outcomes (KDIGO) clinical practice guidelines, is to assess renal function as GFR, along with the serum concentration of endogenous filtration markers. It is suggested to express the GFR as eGFR in milliliters per minute per 1.73 m^2^ rather than milliliters per minute. Additionally, two-stage tests are recommended: an initial test followed by confirmation tests when necessary. Cr-based eGFR is the recommended initial test, while confirmation tests such as Cys C-based eGFR, Cr-Cys C-based eGFR, or CrCl are indicated in specific cases where Cr-based eGFR has evident limitations [60].

The equations used to estimate GFR allow it to be calculated from serum concentrations of endogenous filtration markers, eliminating the need for 24 h urine collection and direct measurements in urine. These markers include low-molecular-weight metabolites (such as Cr, 113 Da) and low-molecular-weight proteins (such as CysC, 1000–20,000 Da).

The serum concentration of these markers is inversely related to GFR. It is also influenced by other physiological factors, known as “non-GFR-related determinants,” which include their generation, renal tubular handling (reabsorption and secretion), and extrarenal elimination. For clinical practice, a specific equation is chosen for each marker or a combination of these to routinely report GFR [62]. See Table 2.

The combination of Cr and CysC for GFR calculation emerged because the race factor was removed to prevent discrimination.

The recalibrated MDRD equation calculates GFR adjusted for body surface area, considering SCr, age, and gender but not ethnicity or muscle mass. Therefore, in individuals with higher muscle mass, this equation often underestimates the actual GFR [63].

## 8. Limitations of Serum Creatinine

Due to Cr’s complex metabolism, measuring eGFR has various limitations. One of the main limitations is that changes in muscle mass, such as in patients with chronic diseases, malnutrition, or advanced age, lead to an overestimation of the GFR.

It is also important to note that all formulas for calculating the GFR do not consider other common factors, such as the intake of cooked red meat and the effect that some medications may have.

Creatinine may retain its utility in usual clinical scenarios. For instance, in individuals with stable renal function and without confounding factors such as recent changes in muscle mass or medication interference, creatinine measurement may still serve as a reliable marker of kidney function.

## 9. Acute Increase in Serum Creatinine

An increment in serum Cr concentration mostly means impaired kidney function. It is expected in patients affected by many chronic illnesses. The silent start and progress of CKD in many of those diseases, such as diabetes, hypertension, as well as some primary kidney diseases, is the cause of the delayed diagnosis of CKD. It is “suddenly discovered” during a routine exam or when secondary manifestations are in study, such as anemia, fatigue, and weight loss.

Actual “acute” increments of serum Cr may occur after well-identified recent events, such as accidents with hemorrhage or severe muscle damage, exercise with excessive dehydration, toxins or poisons, and pain from stones descending into the urinary tract. Under such conditions, differential diagnosis for Cr increments is necessary [64].

To distinguish if increased Cr levels mean acute kidney injury (AKI), increments ≥0.3 mg/dL (≥26.5 μmol/L) within 48 h or an increase in SCr to ≥1.5 times over baseline values are necessary. Additional data as urine volume < 0.5 mL/kg/h for 6 h, muddy brown casts in the sediment, Uosm (mmol/kg) < 350, mild to moderate proteinuria, UNa (mmol/L) > 40, FENa (%) > 1, FEUrea (%) > 35, and novel biomarkers like KIM-1, cystatin C, and NGAL sustain the AKI diagnosis [65].

AKI syndrome encompasses various etiologies, including prerenal azotemia, which accounts for around 30–60% of cases. The reduction in the renal blood flow decreases the glomerular perfusion pressure, resulting in a lower glomerular filtration rate. Because creatinine excretion mainly depends on glomerular filtration, this reduction limits its clearance from plasma, causing elevated serum creatinine levels [66]. The ratio of serum BUN to creatinine in healthy individuals is 10 to 15:1 (when both are expressed in mg/dL or 40 to 60 when expressed in mmol/L). In prerenal AKI, the ratio may be greater than 20:1 because of a disproportionate increase in urea reabsorption resulting from elevated serum vasopressin levels. Intrinsic accounts for around 40% (acute tubular necrosis, acute interstitial nephritis, acute glomerular, and vasculitic renal diseases). In intrinsic AKI, the plasma membrane ruptures, releasing its cytoplasmic content, exacerbating inflammation and injury, reflected as a rise in SCr [67]. Acute postrenal obstructive nephropathy accounts for around 10% [68]. In postrenal AKI, hemodynamic changes combined with obstruction impede urine excretion and result in elevated blood creatinine levels [69].

### Reflection Points

1. Acute increase in serum creatinine (AKI) is a process in which treatment decisions must be made quickly; despite the problems of Crea interpretation, it remains the diagnostic axis for this disease. Therefore, besides SCr, assessing daily urine volume can narrow down the differential diagnosis, dividing AKI into oliguric (<500 mL) and non-oliguric causes. A careful history, physical examination, and basic laboratory tests often suffice for diagnosis.
2. To improve diagnostic precision and therapeutic outcomes in AKI, a shift is needed from SCr-based staging alone to a more comprehensive approach. This includes integrating biomarkers, transcriptomic and proteomic data, and considering the pathophysiological and anatomical context of kidney injury [70,71].
3. Despite its drawbacks, which have already been mentioned, SCr remains the most widely used biomarker in the diagnosis of CKD due to its low cost and rapid analysis in hospitals with limited resources. In these cases, the recommendation is to consider the clinical severity of the disease and recent changes in food consumption and medication for a better interpretation of eGFR.

## 10. Cystatin C

CysC is a proteinase inhibitor belonging to family 2 of the cystatin superfamily. It consists of a no glycosylated polypeptide chain of 120 amino acid residues, it has a molecular weight of 13.3 kDa, is positive charged, and has an isoelectric point of 9.3.

**Figure 8:**
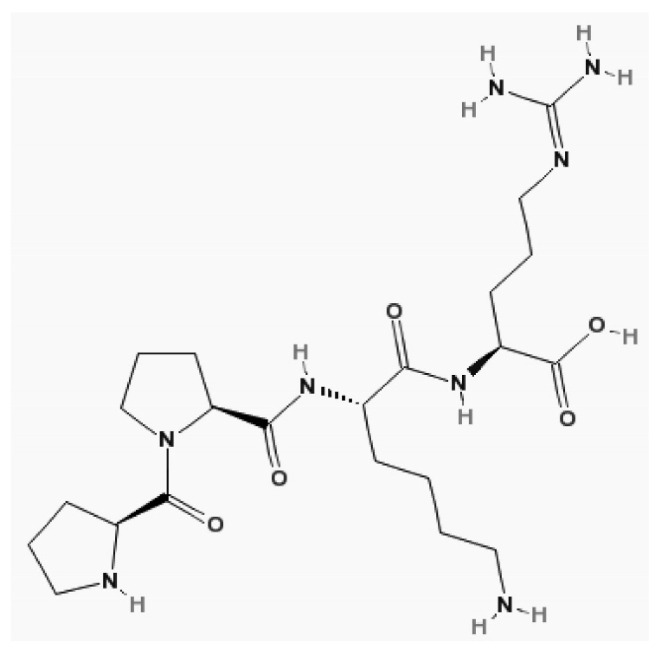
The structure of cystatin C. Molecular formula of cystatin C, C22H40N8O5 [19].

Grubb et al. first determined its structure in 1981 [72]. Later, it was discovered that the human CysC gene, along with its promoter, is of a constitutive type, ensuring its constant production in all nucleated cells [73]. Intracellularly, it acts as a cysteine protease inhibitor, and extracellularly, it acts as a lysosomal proteinase inhibitor [74]. CysC is distributed only in the extracellular volume and cleared by the kidney [75]. Therefore, after acute changes in GFR, the CysC serum concentration increases faster than Cr [76]. Its structure is shown in Figure 8. This molecule is freely filtered by the renal glomerulus under conditions where renal function is not impaired [77]. It is then reabsorbed and fully catabolized by the cells of the proximal tubules, with this absorption occurring through the megalin endocytic receptor. Unlike Cr, it does not undergo tubular secretion [78].

However, it is essential to note that albuminuria can interfere with the process of absorption since CysC and albumin are reabsorbed in the proximal tubule via megalin-facilitated endocytosis [79]. Albuminuria, consequently, increases its excretion in urine due to the competition between the two molecules for the same transport mechanism.

A point in favor of using CysC is that its serum values are not influenced by physiological factors such as age, sex, race, diet, and muscle mass. The study conducted by Chen et al. in the UK Biobank showed that GFR based on CysC identifies five times more participants with a TFG < 60 mL/min/1.73 m^2^ compared to the estimate using only Cr in South Asian Individuals. Furthermore, the use of CysC allowed for the detection of a population with chronic kidney disease who were at high risk of death, heart failure, and cardiovascular atherosclerotic diseases, risks that were not identified when using Cr as a marker. Additionally, data from the UK Biobank study showed that, unlike Cr, CysC can assess kidney function independently of ethnic origin [80].

## 11. Factors Affecting Serum Cystatin C Levels

Pathological factors, such as hyperthyroidism and hypothyroidism, can affect serum CysC levels. It has been shown that in untreated hyperthyroid patients, serum CysC levels are higher than in euthyroid patients, and CysC levels decrease once appropriate treatment is initiated. In addition, in hypothyroid patients who were not receiving any treatment, the serum CysC levels were lower than in euthyroid patients, and the CysC levels increased after receiving the appropriate treatment [81,82,83].

One of the main advantages is that they are less susceptible to interference from factors that affect the measurement of Cr, such as hemolysis, lipemia, and jaundice, providing a more reliable assessment of renal function [84].

Although it has been shown that CysC concentrations can be influenced by pathological conditions such as obesity, inflammation, smoking, steroid treatments, and thyroid disorders this marker is not significantly influenced by physiological factors. Unlike Cr, CysC offers a more reliable and consistent measurement of renal function. CysC is a better tool for assessing renal and cardiovascular risk across different populations, ensuring greater consistency in different clinical settings.

Its measurement is typically carried out using turbidimetric (PETIA) or nephelometric (PENIA) immunoassays. This is because PETIA and PENIA provide several advantages, including fast processing times, minimal interference from other substances, and improved accuracy.

The different characteristics of Cr and CysC are summarized in Table 3.

## 12. Practical Importance of Using the Difference (eGFR Cys C–eGFR Cr) or the Ratio (eGFR Cys C/eGFR Cr)

The use of Cr or CysC to estimate the GFR is not exclusive; these should be considered complementary when evident extrarenal factors may expressly limit the use of some of the biomarkers, as is the case of sarcopenia or thyroid diseases [88]. In most patients, there is concordance in eGFR based on either of the two methods. However, eGFR based on both equations is more reliable [60].

In recent years, evidence has been provided that the difference (eGFR Cys C–eGFR Cr) or the ratio (eGFR Cys C/eGFR Cr) of the two measurements has practical importance. Furthermore, they have a better predictive value for mortality than separate measurements [89]. The discrepancy between the two methods has been attributed to reduced glomerular permeability for proteins with molecular weight in the CysC range. It is known as selective glomerular hypofiltration syndrome (SGHS) [90] or known tentatively as Shrunken Pore Syndrome (SPS) [88]. Usually, these alterations are not detected in isolated evaluations of eGFR and may be present even in patients who do not meet the CKD criteria with current clinical practice guidelines. Ratios (eGFR Cys C/eGFR Cr) less than 0.70 or significant differences (eGFR Cys C–eGFR Cr) are associated with more significant kidney damage and higher mortality rates in various clinical conditions. This can affect people with pre-eclampsia/eclampsia, cardiothoracic diseases and surgery, older adults, and even healthy populations.

Serum creatinine levels should be used for the initial assessment of the GFR in detecting and staging AKI and CKD, determining CKD progression, and making treatment decisions, including those related to kidney replacement therapy [60].

Table 4 outlines the clinical indications for using eGFR calculated with creatinine, cystatin C, or a combination of both. For instance, a high-protein diet may underestimate eGFR when calculated using creatinine alone. In cases of malnutrition, reduced muscle mass can lead to an underestimation of eGFR when relying solely on SCr. Therefore, in patients with cachexia or sarcopenia, it is recommended to use a combination of creatinine and cystatin C to assess eGFR. Similarly, in patients using steroids, due to their effects on muscle mass, eGFR calculated with both markers (Cr-Cys) is suggested. In cases where medications reduce tubular secretion, it is recommended to use eGFRcys [60].

## 13. Utility of Creatinine as Body Composition Marker

### 13.1. Body Composition

Body composition evaluations include evaluations at different levels of organization, from the subcellular and cellular level to the whole body. From a clinical perspective, human body composition can be divided into two main compartments: water compartments and fat mass. Lean mass and cellular mass are calculated based on water compartments and are influenced by age, sex, and race.

Body composition measurements can be performed using different technologies, such as isotopic dilution, computed tomography, magnetic resonance imaging, X-ray densitometry (DEXA), and other more sophisticated technologies. However, indirect, low-cost, and non-invasive methods in clinical practice are commonly used to determine human body composition, such as bioelectrical impedance (BIA) and skinfold thickness. Muscle mass is highly variable between the elderly and children and can be substantially modified through physical exercise, as previously mentioned [55].

### 13.2. Bioimpedance

BIA is a method for estimating body composition by conducting a low-intensity multifrequency current through the human body. Higher frequencies (>50 kHz) are transmitted through the water into the cells and extracellular space, and low frequencies (<50 kHz) are transmitted through water only in the extracellular spaces. Fat mass is more resistant than non-fat cells.

Intracellular water is calculated based on total body and extracellular water differences. Lean body mass is calculated from intracellular water, assuming a constant intracellular water content, usually around 70% [91]:

Total body water = Extracellular Water + Intracellular Water

Lean body mass = ICW/0.70

In dialysis patients, the relationship between compartments is abnormal due to muscle wasting and intracellular and extracellular edema. Therefore, BIA measurements show an increased ICW and overestimate LBM because ICW is greater than 70% (used as a reference in normal subjects). This presumed edema introduces a systematic error in the calculation of LBM. An alternative method is necessary to assess how much BIA overestimates LBM. Among other alternative methods is Cr kinetics, which measures only muscle mass. However, information on this is very scarce.

### 13.3. Creatinine Kinetics and Creatinine Index

Cr generation is an indirect measure of muscle mass because muscle metabolism is the main source of Cr production. Mitch y col [4] conducted a study decades ago on creatine metabolism in patients with CKD, specifically the production and excretion of Cr

Despite its utility in inferring muscle mass, they observed that Cr excretion did not fully explain SCr levels. They concluded that part of this Cr is metabolized endogenously. As SCr increased, creatine was recycled rather than excreted in the urine. Therefore, the need arose to consider the complete Cr metabolism in estimating LBM. Later, Keshaviah [92] defined a formula for calculating LBM in patients on peritoneal dialysis (PD), which is still used today.

The kinetics of Cr are based on the principle that Cr generation is proportional to LBM in patients with constant protein consumption. This technique considers the sum of Cr excretion and metabolic degradation. Cr excretion includes only urinary excretion in non-dialysis patients and Cr content in drained dialysis solutions, plus urinary excretion obtained from residual renal function. At the same time, metabolic degradation is proportional to body weight and SCr concentrations. Therefore, the sum of degradation and excretion should, in a steady state, equal the Cr production rate (Table 5) [4,92].

Subsequently, Bhatla B et al. [93] correlated measurements of LBM using Cr kinetics, bioimpedance, and DEXA in patients with PD and found a high association. Cr kinetics have also been used to assess protein nutritional status in patients with HD [94]. Recently, the same authors found that the CI or Cr kinetics are surrogate tools for measuring muscle mass in patients with kidney damage [95] and added urea distribution to the formula in patients on HD. Since then, no studies have referred to this method in patients with PD.

Recently, Cr kinetics were recommended in the KDOQI (Kidney Disease Outcomes Quality Initiative) guidelines to measure muscle mass in patients with CKD [96]. However, the procedure has the disadvantage that it requires a 24 h urine collection and drained PD solutions, which can sometimes be difficult for the patient. Additionally, meat consumption and creatine supplements increase UCrE, which must be accounted for when calculating Cr kinetics. In anuric HD patients, Cr kinetics are based on the SCr levels before and after HD.

The methods to calculate and use the CI according to the KDOQI guidelines are highlighted in Table 6 [5]:

Cr kinetics’ utility in assessing nutritional status has been questioned; it is not accepted that UCr is associated with muscle mass [97]. On the other hand, such an association is accepted as long as the subjects have no nutritional restrictions and 24 h urine was collected over three consecutive days [98]. These two studies were conducted in healthy subjects; however, in patients with kidney damage, such as those on PD, Cr excretion may be inaccurate since it does not consider recycling and Cr returns to circulation, which is why these patients have high SCr values. It is also necessary to consider the losses of Cr in the drained PD solution that the patient should collect.

BIA and creatinine kinetics are useful tools for assessing body composition, though each has limitations. Fluid imbalances can influence BIA, while creatinine kinetics require careful urine collection. Despite these challenges, creatinine kinetics provide a more reliable estimate of lean mass, particularly in patients with kidney issues, making it a valuable clinical tool.

Measurement of LBM is important in the nutritional management of patients to quantify sarcopenia, energy waste, and mortality outcomes.

### 13.4. Conclusions and Future Directions

Creatinine measurements are an easy and widely available technique for routinely estimating the glomerular filtration rate and muscle mass. Despite its utility in daily clinical work, the medical team should know its limitations and be aware of misinterpretation. Serum creatinine should be used for the initial assessment of GFR in detecting and staging acute kidney disease and CKD. Alternative or complementary measurements of cystatin C must be considered when evaluating kidney function, including dosing medications and making treatment decisions. However, the utility of Cr is good when using Cr kinetics as a direct measurement of lean body mass compared to electrical bioimpedance, which underestimates muscle mass in patients with edema, such as those with CKD.

## Figures and Tables

**Table 1:** Creatine in foods [9].

Herring	8.0 g/kg
Red meats (Beef, veal, Lamb)	5.0 g/kg
Pork	5.0 g/kg
Liver	4.5 g/kg
Salmon	4.5 g/kg
Tuna	4.0 g/kg
Chicken	4.0 g/kg
Rabbit	3..5 g/kg
Cod	3.0 g/kg
Soy	2.0 g/kg

**Table 2:** Formulas to determine renal function.

	Formula
**CrCl**	= UCr (mg/dL) × (Urine vol. 24 h mL/(1440 min)/SCr (mg/dL)
**Cockcroft–Gault**	= [(140 − age) × weight in kg]/(72 × SCr × 0.85 (women)
**MDRD 4**	= (175 × SCr^−1.154^) × (age^−0.203^) × 1 (men) = (175 × SCr^−1.154^) × (age^−0.203^) × 0.742 (women)
**MDRD 6**	= (170 × SCr^−0.999^) × (age^−0.176^) × (BUN^−0.17^) × (albumin^0.318^) × 1 (men) = (170 × SCr^−0.999^) × (age^−0.176^) × (BUN^−0.17^) × (albumin^0.318^) × 0.762 (women)
**CKD-EPI**	= 141 × (SCr/0.9)^−1.209^ × (0.993^age^) (men) = 144 × (SCr/0.9)^−1.209^ × (0.993^age^) (women)
**CKD-EPI CysC**	= 133 × (sCysC/0.8)^−1.328^ × (0.996^age^) (men) = 133 × (sCysC/0.8)^−1.328^ × (0.996 ^age^) × 0.932 (women)
**CKD-EPI CrCysC**	= 135 × (SCr/0.9)^−0.601^ × (sCysC/0.8)^−0.711^ × (0.995^age^) (men) = 130 × (SCr/0.7)^−0.601^ × (sCysC/0.8)^−0.711^ × (0.995^age^) (women)

MDRD = modification of diet in renal disease; CKD-EPI = Chronic Kidney Disease Epidemiology Collaboration; CKD-EPI CrCysC = Chronic Kidney Disease Epidemiology Collaboration equation using both serum creatinine and cystatin C; CrCl = creatinine clearance; UCr = urinary creatinine; SCr = serum creatinine; CysC = cystatin C; sCysC = serum cystatin C [60].

**Table 3:** Comparison of serum creatinine and cystatin C.

	Creatinine	Cystatin C
**Renal processing**	Excreted by the kidneys, mainly via glomerular filtration. It undergoes tubular secretion [19].	Freely filtered by the glomeruli. Reabsorbed and catabolized by renal proximal tubular cells. It does not undergo tubular secretion [78].
**Factors that decrease it in serum**	South Asian patients, aging, female sex, neuromuscular disease, malnutrition, and hyperthyroidism [50,85].	Hypothyroidism [81,82,83].
**Factors that increase it in serum**	Black ethnicity, higher muscle mass, rhabdomyolysis, high-protein diet, supplements, steroid treatment, and various medications [3,10,11,28,49,55].	Chronic inflammation, obesity, hyperthyroidism, and chronic smoking [86].
**Price and processing time**	USD 1; 15 min.	USD 10; 1 h.
**Advantages**	Easier and more widely available technique. Healthcare professionals are more comfortable with its use.	Less influenced by diet, physical activity, or physiological factors [87].
**High risk of death, heart failure, and cardiovascular atherosclerotic diseases—risks**	It does not identify them.	It identifies them [60].

**Table 4:** Clinical indications for using creatinine or cystatin C [60].

Clinical Indications	Recommendations
**Body Habitus and Changes in Muscle Mass, Extreme Sport/Bodybuilder**	eGFRcys if there are no comorbidities.
**Above-knee Amputation**	eGFRcys if there are no other comorbidities. eGFRcr-cys if comorbidities exist.
**Obesity Class III**	Use eGFRcr-cys.
**Lifestyle Factors** **(Low-protein diet, keto diet, vegetarian, high-protein diet, etc.)**	eGFRcr if no changes to non-GFR determinants of SCr or no comorbid illness.
**Malnutrition**	eGFRcr-cys may be less accurate, use mGFR.
**Muscle Wasting Diseases**	eGFRcr-cys for routine evaluation.
**Steroids (Anabolic, Hormone)**	Use eGFRcr-cys.
**Medications effects** **(Tubular Secretion Decrease)**	eGFRcys if there are no other comorbidities.
**Acute Kidney Injury**	Use eGFRcr.
**Initial approach to Glomerular Filtration Rate**	Use eGFRcr.

**Table 5:** Formulas used in creatinine kinetics [4,92].

	Formula
**Fat free mass**	= 0.029 × Creatinine Production (mg/day) + 7.35
**Creatinine Production (mg/day)**	= Excretion + Metabolic Degradation
**Creatinine Excretion (mg/day)**	= (Urine Volume) × (Urinary Creatinine) + (Dialysis Volume) × (Creatinine in Dialysis Liquid)
**Metabolic Degradation (mg/day)**	= 0.38 × Serum Creatinine (mg/dL) × Total Body Weight (kg)

**Table 6:** Formulas to calculate the creatinine index.

	Formula
**Edema-free lean body mass (kg)**	= (0.029 kg/mg/24 h) × creatinine index (mg/24 h) + 7.38 kg
**Creatinine index (mg/24 h)**	= Dialysate (or ultrafiltrate) × creatinine (mg/24 h) + urine creatinine (mg/24 h) + change in body creatinine pool (mg/24 h) + creatinine degradation (mg/24 h)
**Change in body creatinine pool** **(mg/24 h)**	= Serum creatinine (mg/dL)_f_ − [serum creatinine (mg/dL)_i_)] × [24/h/(time interval between the i and f measurements)] × [body weight (kg) × (0.50 L/kg)]
**Creatinine degradation rate (mg/24 h)**	= 0.38 dL/kg/24 h × serum creatinine (mg/dL) × Body weight (kg)

## Data Availability

Not applicable.

## Abbreviations

AASK	African American Study of Kidney Disease and Hypertension
ADP	Adenosine diphosphate
AGAT	L-arginine amino transferase
Arg	Arginine
ATP	Adenosine triphosphate
BIA	Bioelectrical Impedance Analysis
BMI	Body mass index
CI	Creatinine index
CK	Creatine kinase
CKD	Chronic kidney disease
CKD-EPI	Chronic Kidney Disease Epidemiology Collaboration
Cr	Creatinine
CrCl	Creatinine clearance
Crn	Creatine
CysC	Cystatin C
Da	Dalton
DTG	Dolutegravir
DEXA	Dual-Energy X-ray Absorptiometry
eGFR	Estimated glomerular filtration rate
ECW	Extracellular fluid
ESPMT	N-ethyl-N-sulfopropyl-m-toluidine
FFM	Fat-free mass
GFR	Estimated glomerular filtration rate
GAA	Guanidinoacetic acid
GAMT	S-adenosyl-L-methionine methyltransferase
Gly	Glycine
GFRcys	Estimated glomerular filtration rate from cystatin C
GFRcr	Estimated glomerular filtration rate from creatinine
GFRcr-cys	Estimated glomerular filtration rate from creatinine and cystatin C
HD	Hemodialysis
ICW	Intracellular fluid
kDa	Kilodalton
KDIGO	Kidney Disease Improving Global Outcomes
KDOQI	Kidney Disease Outcomes Quality Initiative
LBM	Lean body mass
MATE1	Multidrug and toxin extrusion protein 1
MATE2-K	Kidney-specific multidrug and toxin extrusion protein 2
MDRD	Modification of diet in renal disease
NADH	Nicotinamide adenine dinucleotide
NGAL	Neutrophil gelatinase-associated lipocalin
NHANES	National Health and Nutrition Examination Survey
OAT2	Organic anion transporter 2
OAT3	Organic anion transporter 3
OCT2	Organic cation transporter 2
OCT3	Organic cation transporter 3
PD	Peritoneal dialysis
SAM	S-adenosylmethionine
SCr	Serum creatinine
TBW	Total body water
TDF	Tenofovir disoproxil fumarate
UCr	Urinary creatinine concentration
UCrE	Urinary creatinine excretion
UK Biobank	UK Biobank Health Database
UV	Ultraviolet
μM	Micromolar
