# Correction: Ávila et al. The Metabolism of Creatinine and Its Usefulness to Evaluate Kidney Function and Body Composition in Clinical Practice. *Biomolecules* 2025, *15*, 41

**DOI:** 10.3390/biom15121719

**Published:** 2025-12-11

**Authors:** Marcela Ávila, Mariana G. Mora Sánchez, Alma Sofía Bernal Amador, Ramón Paniagua

**Affiliations:** Unidad de Investigación Médica en Enfermedades Nefrológicas, Hospital de Especialidades, CMN SXXI, Instituto Mexicano del Seguro Social, Ciudad de México 06720, Mexico

In the original publication [1], the citation of Reference 9 has now been changed in Section 3.1.1 in the first paragraph:

“Exogenous Crn primarily comes from the diet and its processing. Some of the main foods where Crn can be found are shown in Table 1 [9].”

**Table 1.** Creatine in foods [9].

Therefore, the following reference is now:

9. Balsom, P.D.; Söderlund, K.; Ekblom, B. Creatine in humans with special reference to creatine supplementation. *Sports Med.* **1994**, *18*, 268–280.

In order to keep a consistent format, the citation of Reference 60 was moved to Table 4 caption, and now it reads:

**Table 4.** Clinical indications for using creatinine or cystatin C [60].

Reference 4 was not cited in Table 5 and has now been inserted in Section 13.3 Creatinine Kinetics and Creatinine Index in the fourth paragraph, as well as Caption of Table 5 and should now read:

“[…] Therefore, the sum of degradation and excretion should, in a steady state, equal the Cr production rate (Table 5) [4,92].”

**Table 5.** Formulas used in creatinine kinetics [4,92].

The multiplication signs in Tables 2, 5 and 6 were also updated.

The authors state that the scientific conclusions are unaffected. This correction was approved by the Academic Editor. The original publication has also been updated.
